# Editorial: The Pivotal Role of Oral Microbiota Dysbiosis and Microbiota-Host Interactions in Diseases

**DOI:** 10.3389/fcimb.2022.947638

**Published:** 2022-06-14

**Authors:** Xin Xu, Jin Xiao, Yulong Niu

**Affiliations:** ^1^ State Key Laboratory of Oral Diseases, National Clinical Research Center for Oral Diseases, West China Hospital of Stomatology, Sichuan University, Chengdu, China; ^2^ Eastman Institute for Oral Health, University of Rochester Medical Center, Rochester, NY, United States; ^3^ Key Laboratory of Bio-Resources and Eco-Environment of Ministry of Education, College of Life Sciences, Sichuan University, Chengdu, China

**Keywords:** oral diseases, dental caries, periodontitis, oral microbiota, probiotics, HIV - human immunodeficiency virus, Raman spectoscopy

The oral microbiome is an important constituent of human microbiome, playing a pivotal role in human health. Oral microbial dysbiosis is the major causative factor of oral diseases such as dental caries and periodontal diseases, and it is also closely associated with systemic diseases such as cardiovascular diseases, diabetes, and gastrointestinal diseases, etc (Hajishengallis, 2015; Hajishengallis and Chavakis, 2021). The last two decades have witnessed tremendous progress in the field of oral microbiota and its related human diseases, largely due to the advancement in high throughput “-omics” techniques such as metagenomics and metatranscriptomics. In addition, the development of non-invasive detection methods such as Raman Spectroscopy also makes the dynamic detection of microbial metabolic activity possible (Su et al., 2020). Oral microbiota can be recognized as the “fingerprint” of human health, and tools and models developed based on metadata and comprehensive bioinformatics can be utilized to robustly predict the occurrence and prognosis of oral diseases as well as the related systemic diseases (Xu et al., 2017). In addition, identification of *Porphyromonas gingivalis* as the keystone pathogen of periodontitis has greatly advanced the knowledge on the pathogenesis of this disease (Hajishengallis et al., 2012). Novel approaches that can specifically target key pathogens are promising for the ecological management of oral diseases. In this Research Topic, we have included eight original research articles as well as six comprehensive reviews, covering the novel findings in oral microbiota dysbiosis and microbiota-host interactions, and new compounds or novel approaches in the diagnosis and treatment of diseases associated with oral microbial dysbiosis.

The relationship between oral cancer and microbiome has been suggested, but with controversial conclusions (Kamarajan et al., 2020; Sepich-Poore et al., 2021). Chen et al. investigated the salivary microbiome in the cohorts of orally healthy, non-recurrent oral verrucous hyperplasia, and oral verrucous hyperplasia–associated oral cancer at taxonomic and function levels. They demonstrated that predicted functional profiles were more related to the alterations of oral health status as compared to taxonomic data. In addition to oral cancer, increasing evidence has suggested the association of HIV infection and oral microbiota (Annavajhala et al., 2020; Fulcher, 2020). However, the impact of antiretroviral therapy (ART) on oral microbiome of HIV-infected patients has yet to be investigated. Li et al performed a longitudinal comparative study, and investigated the oral microbial alterations in the men who have sex with men with acute/chronic HIV infections. They found that microbial diversity was significantly decreased in patients with acute and chronic HIV infections compared with those HIV-free individuals before and after ART. Specific genera with altered abundance were also identified to be associated with HIV infection and ART administration. In addition to bacterial dysbiosis, HIV-infected individuals are more susceptible to fungal infections (Patil et al., 2018). The longitudinal study by Chang et al. identified an increased diversity and richness of salivary mycobiome in HIV-infected individuals as compared to the HIV-free controls. After ART, the diversity and richness of salivary mycobiome in HIV-infected patients were reduced significantly. These findings suggest that both HIV infection and ART administration have significantly impact on salivary mycobiome, which might mirror the immune state of the body. In addition to viral infection that can alter oral microbiome, translocation of specific pathogens also contributes to the microbial alterations in the oral cavity. A cross-sectional study on reflux esophagitis patients by Liang et al. demonstrated that reflux esophagitis significantly disturbed oral microbiome with an increased beta diversity, and *Helicobacter pylori* infection could inhibit this disorderly trend.

As the occurrence, progression and prognosis of oral diseases are accompanied with compositional and metabolic alterations of oral microbiota, development of non-invasive, high throughput detection tools with single-cell resolutions are promising in the diagnosis, treatment planning and outcome evaluation of oral diseases. Raman spectroscopy detects molecular vibration information by collecting inelastic scattering light, which provides rapid, sensitive, accurate, and minimally invasive detection (Xu et al., 2017). Zhang et al. reviewed the application of Raman spectroscopy in the early diagnosis, treatment and prognosis evaluation of oral diseases including dental caries, periodontal diseases and oral cancer. Although Raman spectroscopy is promising, the authors suggested that future efforts to increase signal-to-noise ratios and develop robust tools for data analysis are still needed.

Bacterial protein phosphorylation systems have been suggested to be involved in microbial dysbiosis and microbes-host interaction (Lamont et al., 2018). Ren et al. discussed the roles of tyrosine and serine/threonine phosphorylation systems in keystone species *P. gingivalis*, with a particular focus on their involvement in bacterial metabolism and virulence, community development, and bacteria-host interactions. In addition to its association with periodontitis, *P. gingivalis* can increase the risk of systemic diseases such as type 2 diabetes mellitus (T2DM), cardiovascular diseases, nonalcoholic fatty liver disease (NAFLD), rheumatoid arthritis, and gut inflammation (Hajishengallis, 2015; Kitamoto et al., 2020; Hajishengallis and Chavakis, 2021). Previous study by Xu’ group demonstrated that *P. gingivalis* was able to induce insulin resistance by increasing the serum level of branched-chain amino acid (BCAA) in high fat diet (HFD)-fed mice (Tian et al., 2020). Work by Wu et al. further demonstrated that *P. gingivalis* elevated serum level of BCAA and exacerbated liver injury in HFD-fed mice, and this effect was dependent on the bacterial BCAA transport system genes livh/livk.

*Streptococcus mutans* is the main aciduric and acidogenic species in the oral cavity, and its persistence within the multispecies oral biofilms may antagonize commensal bacteria and drive compositional shift of oral microbiota towards a more cariogenic community that favors the development of dental caries (Lamont et al., 2018; Du et al., 2021). Fluoride is an effective anti-caries agent. However, widespread application of fluoride may induce fluoride-resistance in *S. mutans* (Li et al., 2021). Zhang et al. established an antagonistic dual-species biofilm consisting of *S. mutans* and *Streptococcus sanguinis*, and demonstrated that fluoride-resistant strain of *S. mutans* gained a survival advantage over *S. sanguinis* with an excessive production of extracellular polysaccharides after fluoride exposure, challenging the control of dental plaque biofilm. Dental caries is a multifactorial disease, and its progression is closely associated with ecological shift of oral microbiota towards a more cariogenic community that favors the demineralization of tooth hard tissue (Lamont et al., 2018). Wu et al. created a multifactorial machine learning model using oral microbiome of mother-child dyads in combination with demographic-environmental factors and relevant fungal information. By using this model, they identified specific caries-associated oral bacteria, *Candida*, and other multi-source factors for preschool children and their mothers.

The interactions between host and microbes play pivotal role in the development of oral diseases. Osteomicrobiology is a novel terminology that refers to the role of microbiota in bone homeostasis. Recent studies have shown the roles of oral microbiota in modulating host defense systems and alveolar bone homeostasis. Cheng et al. proposed the terminology “oral osteomicrobiology” and discussed the regulation of alveolar bone development and bone loss by oral microbiota under physiological and pathological conditions. Signaling pathways involved in oral osteomicrobiology and critical techniques for related investigations were also introduced. The recognition of pathogen-associated molecular patterns (PAMP) or damage-associated molecular patterns (DAMP) by the pattern-recognition receptors (PRRs) such as Toll-like receptors (TLRs) and nucleotide-binding oligomerization domain-like receptors (NLRs) has been well documented as the main molecular mechanisms for the host-microbial interactions (Akira et al., 2006; Kanneganti et al., 2007). Recent studies have identified a wide expression of extra-gustatory taste receptors in tissues/organs including airways, nasopharyngeal cavities, gastrointestinal tract and gingivae, and demonstrated their pivotal role in host immune responses and infectious diseases (O'Leary et al., 2019; Schneider et al., 2019; Ting and Von Moltke, 2019; Zheng et al., 2019). A review article from Dong et al discussed how taste receptors, particularly bitter and sweet taste receptors, mediated the oral microbiota-host interaction and the development of oral diseases. The taste receptor-mediated signaling and host immune responses may provide novel treatment targets for the management of oral infectious diseases such as periodontal diseases.

Since frequent use of wide-spectrum antimicrobials may cause microbial dysbiosis and drug resistance, novel approaches that can restore microecology without necessarily killing the bacteria are promising (Kuang et al., 2018). Zhang et al. reviewed the application of probiotics in the management of periodontal diseases. Probiotic bacteria derived from the genera *Lactobacillus*, *Bifidobacterium*, *Streptococcus*, and *Weissella* have shown effectiveness in the prevention and treatment of periodontal diseases. Competition for adhesion sites, antagonism against growth, biofilm formation and virulence expression of periodontopathogens, and regulation on host immune responses are the most recognized mechanisms. Nevertheless, more well-controlled clinical trials are still needed to provide solid evidence for their clinical usage. Small molecules which can either selectively inhibit keystone microbes or suppress the key virulence of the microbial community, are promising for the ecological management of oral diseases. Yang et al. discussed the research progress in the development of antimicrobial small molecules and delivery systems, with a particular focus on their antimicrobial activity against typical species such as *S. mutans*, *P. gingivalis* and *Candida albicans*. Although future work is still needed to delineate its molecular mechanisms and the exact drug targets, the authors believed that small molecules with potent antimicrobial activity, high selectivity, and low toxicity are promising for the ecological management of oral diseases. In addition to probiotics and small molecules, poly(amidoamine) dendrimers with amino terminal groups (PAMAM-NH2) have been identified as promising antimicrobial agents (Mintzer et al., 2012). Secondary caries caused by microbial leakage and hybrid layer degradation is one of the major causes of treatment failure of dental caries. Gou et al. developed a novel dentin cavity cleanser that contains PAMAM-NH2. Although the exact molecular mechanisms are still unclear, the PAMAM-NH2 showed long-term antimicrobial and anti-proteolytic activities, which are crucial for the maintenance of resin-dentin bond durability and thus promote the prevention of secondary caries.

A recurring theme in the current topic is the microbial diversity of oral microbiota and its association with oral and systemic health status. Although we have seen several submissions with longitudinal studies that showed dynamic microbial alterations during the treatment process, the key factors and underlying mechanisms that drive the microbial shift have yet to be investigated. In addition, although functional profiles have been suggested to be more related to diseases as compared to taxonomic alterations, current submissions mainly include data obtained from 16S and 18S rRNA amplicon sequencing. Future studies with metagenomic, meta-transcriptomic and meta-metabolomic approaches are still needed to better delineate the robust interactions between host and microbiota, and thus provide molecular basis for the development of new diagnostics and treatment modalities that target keystone pathogens in oral diseases.

## Author Contributions

XX drafted the editorial and JX, YN contributed to the final submitted version. All authors contributed to the article and approved the submitted version.

## Funding

This work was supported by a grant from Health Commission of Sichuan Province (21PJ058).

## Conflict of Interest

The authors declare that the research was conducted in the absence of any commercial or financial relationships that could be construed as a potential conflict of interest.

## Publisher’s Note

All claims expressed in this article are solely those of the authors and do not necessarily represent those of their affiliated organizations, or those of the publisher, the editors and the reviewers. Any product that may be evaluated in this article, or claim that may be made by its manufacturer, is not guaranteed or endorsed by the publisher.
